# Anxious behaviour in a demonstrator affects observational learning

**DOI:** 10.1038/s41598-019-45613-1

**Published:** 2019-06-24

**Authors:** Ida Selbing, Andreas Olsson

**Affiliations:** Division of Psychology, Karolinska Institutet, 171 77 Stockholm, Sweden

**Keywords:** Fear conditioning, Human behaviour

## Abstract

Humans can acquire fear through the observation of others’ (learning models’) threat responses. These responses can be direct responses to aversive stimuli, or anticipatory responses to threats. Most research focuses on learning from observation of direct responses only. Here, we investigated how observational fear conditioning is influenced by a learning model’s typically anxious anticipatory responses. High anxiety individuals often display typically anxious anticipatory behaviour, such as worsened discrimination between safe and unsafe stimuli, characterized by increased threat responses to safe stimuli. We hypothesized that observation of an anxiously behaving model would worsen discriminatory learning. To this end, we developed an observational conditioning paradigm where a learning model was exposed to one safe and one unsafe stimuli. The learning model displayed anticipatory aversion to either to the unsafe stimulus only (Non-Anxious Model group) or to both the safe and unsafe stimuli (Anxious Model group) in addition to reacting directly to an aversive stimulus paired with the unsafe stimulus. Contrary to expectations, discriminatory learning was not worsened in the Anxious Model group compared to the Non-Anxious Model group. Rather, we saw more robust discriminatory learning in the Anxious Model group. The study provides a first step towards understanding the effect of other’s anticipatory responses in general and typically anxious anticipatory responses in particular, on observational fear learning.

## Introduction

Acquiring fear by observing other peoples’ responses can be an effective way of learning to fear^1–3^ and avoid^4^ potential threats. Observational fear learning is also believed to be a common cause in the development of anxiety disorders^3,5,6^, emphasizing its clinical relevance.

We can for instance learn by observing someone’s direct response to an aversive stimulus, such as when being bitten by a dog. Learning from observation of anticipatory responses might however be more common, such as observing the response when someone who is afraid of dogs encounters a dog. Anticipatory responses are not necessarily reliable cues to the observer about what constitutes a real threat. For instance, we expect individuals high in trait anxiety to behave anxiously and react fearfully to both safe and unsafe stimuli^7^. In the present study we examined if and how observational learning from direct aversive responses is influenced by the anxious anticipatory responses of the observed learning model. More specifically, we want to investigate if such typically anxious behaviour would transfer between individuals.

Individuals vary in how well they learn to differentiate between safe and unsafe cues. For instance, individuals suffering from general anxiety disorder, GAD, and post-traumatic stress disorder, PTSD^8–12^ show reduced discriminatory learning between threatening and safe cues, commonly driven by increased fear responses to safe stimuli. Similarly reduced discriminatory learning has also been demonstrated in subclinically anxious individuals^7,13^. In addition, fear learning can vary as a function of external factors, such as the type of stimuli, e.g. fear relevant^14^, and strength, such as shock intensity^15^, of the stimuli used. There is also evidence of wider generalization of learning to negative, as compared to positive, stimuli, believed to reflect a “better safe than sorry” principle^16,17^. In sum, fear responses vary and we can expect to observe typically anxious behaviour, defined as anticipatory fear responses to both safe and threatening cues, not just from high trait-anxious individuals, but also in highly aversive contexts and to phylogenetically fear relevant stimuli (e.g. snakes).

Observational fear conditioning is studied using discriminative fear conditioning paradigms^18–20^ that are conceptually similar to direct fear conditioning paradigms. In these paradigms, a participant observes another individual, the learning model, being repeatedly presented with two conditioned stimuli (CSs). The CS+ is paired with an inherently aversive stimulus, the unconditioned stimulus (US), such as a mild shock, to the learning model, and the CS− is never paired with the US. The participant observes the learning model’s unconditioned response (UR) to the US, but does not experience the US herself (contrary to direct fear conditioning paradigms). Following successful learning, the participant will exhibit a differential conditioned response, CR, i.e. higher response to the CS+ compared to the CS−, in a subsequent test phase where the participant is exposed to the CSs directly. The responses are commonly measured as skin conductance responses (SCRs) or fear-potentiated startle (FPS). FPS is usually considered to be more valence specific^21^ (although this has been contested^22,23^) and more closely tied to the cross-species threat response system^24^. FPS can be used to assess emotional reactivity both to the discrete presentation of a stimulus as well as to the context, which might be of particular importance when studying anxiety^25^.

While the observation of a CS paired with the learning model’s UR clearly indicates the CS−US association, so too could the pairing of a CS with the learning model’s CR, although perhaps less reliably. We can therefore assume that the learning model’s anticipatory response, the CR, when observable, also contributes to learning. Because people tend to avoid direct interactions with previously aversive events^26^, it is reasonable to assume that it is more common to observe others’ fearful anticipatory responses than observing their direct aversive responses (URs). For example, we are probably more likely to observe someone expressing anticipatory threat responses and avoidance of dogs rather than someone reacting to being bitten by a dog. Yet, even though the observation of others’ fearful anticipatory responses are both common and can provide information about the treat or safety of a stimulus, studies of observational fear learning in the last decades have mainly focused on learning through observation of the learning model’s unconditioned responses during direct encounters with the US^3,27^. There does however exist some early studies of observational fear learning in both humans^28^ and non-human animals^2^ that have studied observational learning from the learning model’s CR rather than its UR.

**Figure 1:**
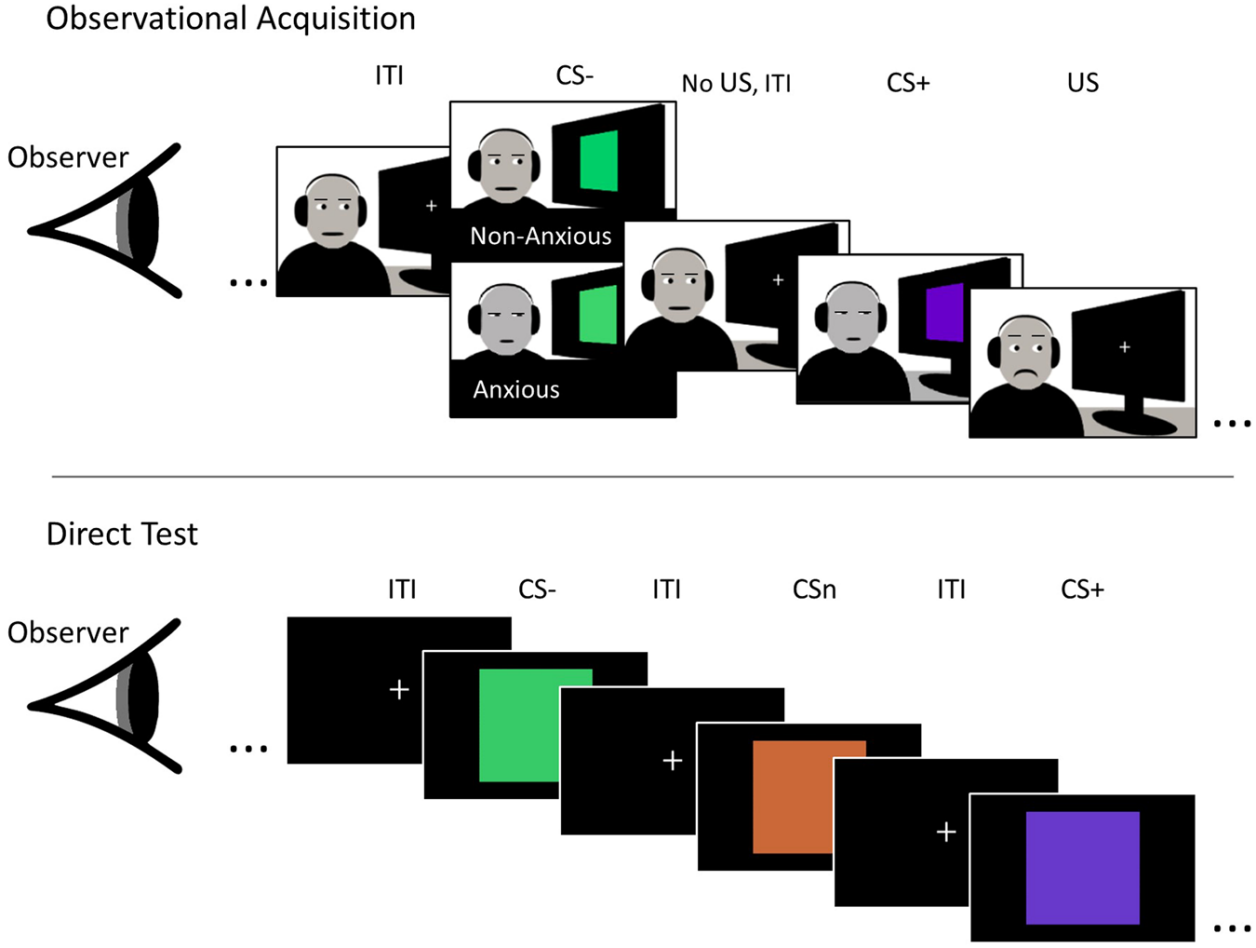
During the observational acquisition, participants observed a video in which a learning model watched a screen were the CS+ and CS− were presented with inter-trial-intervals (ITIs) in between. The anxious model reacted with anticipatory aversion to both the CS+ and the CS−, while the non-anxious model reacted with anticipatory aversion to the CS+ only. Both models reacted aversively when they were presented with the US in the form of an aversive sound in their headphones. During the direct test, participants observed the CS+ and CS−, as well as a novel CS, CSn, directly on the screen. The CSn was included to investigate generalization effects. The observational acquisition was preceded by a pre-acquisition phase where the CS+ and CS− were presented directly on the screen as in the direct test phase.

The main goal of the current study is to investigate if and how observational fear conditioning following exposure to a learning model’s direct response to an aversive stimulus is influenced by the expression of anticipatory anxiety in the learning model, and if this is affected by the observer’s (i.e. the participant’s) trait anxiety. Specifically, we aimed to study if observational conditioning differed as a function of the learning model reacting fearfully to either the CS+ only (non-anxious behaviour) or to both the CS+ and the CS− (anxious behaviour), in addition to reacting aversively to the US. To this aim, participants underwent an observational conditioning procedure^29^ where they first saw a video of a person, the learning model, being presented with a CS+ that was paired with a US and a CS− that was never paired with the US. Participants were divided into two groups. One group observed a learning model that reacted with mild anticipatory fear to the CS+ only (Non-Anxious Model group), whereas the other group observed a learning model that reacted with mild anticipatory fear to both CSs (Anxious Model group). Importantly, in order to be able to study the effects of the learning model’s anticipatory responses to the CS rather than to the UR, the learning model reacted negatively to the US in both groups. Next, observational learning was tested by presenting the CS+ and the CS−, but not the US, to the participants directly. In addition, a novel CS (CSn) was included during the direct test to assess possible generalization effects. (For an overview of the paradigm, see Fig. 1.) Fear responses were measured using fear potentiated startle, FPS, and by subjective ratings of US expectancies. We tested three specific hypotheses. First, since the anxiously behaving learning model did not discriminate between the CS+ and CS−, we expected that participants in the Anxious Model group also would learn to discriminate less efficiently between the CS+ and the CS−. Since the anxious but not the non-anxious model reacted aversively to the CS− we expected that the decrease in discrimination would be due to higher fear responses to the CS−. Second, we hypothesized that participants in the Anxious Model group might generalize fear more to the novel stimulus, CSn, as compared to the Non-Anxious Model group. This would represent an experimental model of the transfer of anxious behaviour, i.e. worsened discrimination, overgeneralization and expressed fear to safe stimuli, from a model to an observer in a real-life situation. Finally, we hypothesized that high trait-anxious individuals might be more susceptible to anxiously behaving learning models and that the effect of observing an anxious learning model would be more pronounced in individuals with higher trait anxiety.

The secondary aim of the study was to develop an observational conditioning paradigm that included an aversive sound as the US. Aversive sounds can be used instead of more traditional types of USs, such as shocks, in populations where these can be ethically problematic, for instance in children or certain clinical populations^30^. We used auditory stimuli from the IADS stimuli set^31^, which includes sounds of varying valence and intensity. In our study, only the learning model experienced the sounds (during filming of the stimulus material), never the participants.

## Results

### Group characteristics

Unpaired t-tests were used to assess possible differences in characteristics between the two groups. The Non-Anxious Model group and the Anxious Model group did not differ in age (*t* = *−0*.2*0*, *df* = *75*.*35*, *p* = *0*.*84*), trait anxiety as measured by STAI-T (*t* = *0*.*14*, *df* = *77*, *p-value* = *0*.*89*; Non-Anxious Model: Mean = 39.3, SD = 7.7, Anxious Model: Mean = 39.6, SD = 7.9) nor empathy as measured by BEES (*t* = *−1*.*08*, *df* = *74*.2*9*, *p-value* = *0*.2*8*; Non-Anxious Model: Mean = 50.0, SD = 24.8, Anxious Model: Mean = 43.2, SD = 30.9). Post-experimental ratings further showed that the groups did not differ in how much they liked (*p* = *0*.*58*) or empathized with (*p* = *0*.*39*) the learning model or how similar to the learning model they judged themselves to be (*p* = *0*.*98*).

### Pre-acquisition

Analyses of expectancy ratings and FPS during pre-acquisition were carried out using linear mixed models (LMMs). There was no significant main effect of CS-type (*p* = *0*.*88*) or Group (*p* = *0*.*10*), or any significant CS-type × Group interaction (*p* = *0*.*53*) on expectancy ratings. Neither was there any significant main effect of CS-type (*p* = *0*.*64*) or Group (*p* = *0*.*16*), or any significant interaction with CS-type or Group (all *ps* > *0*.*57*) on startle responses.

### Observational acquisitio*n*

#### Expectancy ratings

**Figure 2:**
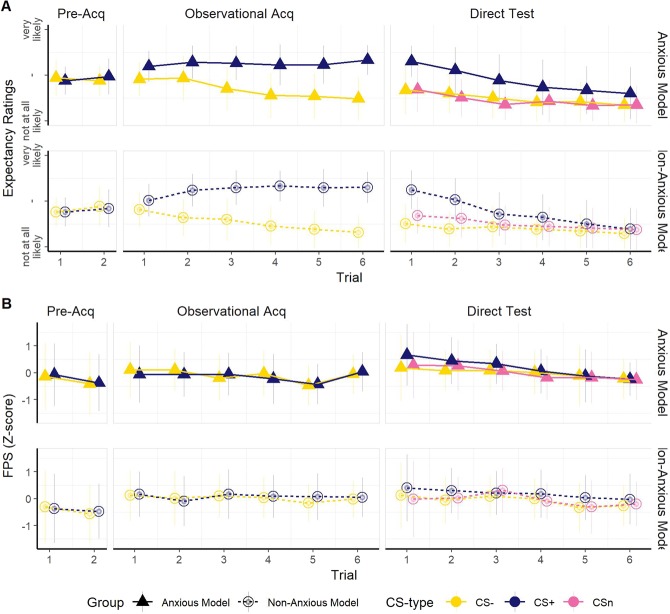
Expectancy ratings and normalized startle responses during pre-acquisition, observational acquisition and direct test. Error bars represent standard deviation of the mean. (**A**) Analyses of expectancy ratings during observational acquisition showed that both groups learned to discriminate equally well between the CS+ and CS−. This was also evident in the direct test phase where however extinction of the differential ratings over trials was slower for the Anxious Model compared to the Non-Anxious Model group. By including the CSn in our analyses we could conclude that there was no difference between groups with regards to generalization. (**B**) Analyses of startle responses showed that participants did not discriminate between the CS+ and CS− during observational acquisition although startle responses were greater during presentation of the CS+ compared to the CS− in the direct test phase, indicating that learning occurred. Including CSn in our analysis did not reveal any signs of differences in generalization between groups.

During the observational acquisition phase, participants continuously rated to what degree they expected that the demonstrator was soon about to hear the aversive sound, the US. Ratings were done using a continuous scale ranging from “not at all likely” to “very likely”. Analyses of these ratings using LMMs revealed higher ratings in the Anxious, as compared to the Non-Anxious, Model group (significant main effect of Group *Χ*^2^(*1*) = *4*.*78*, *p* = *0*.*03*; *β* = *0*. *39*, *SE* = *0*.*18*, *t* = 2.*19*). There were also higher expectancy ratings for the CS+ compared to the CS− (significant main effect of CS-type *Χ*^2^(*1*) = *95*.*41*, *p* < *0*.*001*; *β* = *0*. *93*, *SE* = *0*.2*8*, *t* = *3*.*34*). Furthermore, expectancy ratings for the CS− decreased over trials while they increased for the CS+ (significant main effect of Trial *Χ*^2^(*1*) = *13*.*64*, *p* < *0*.*001;* Trial: *β* = *−0*.*49*, *SE* = *0*.*05*, *t* = *−9*.*3*2; significant Trial × CS-type interaction *Χ*^2^(*1*) = *93*.*7*2, *p* < *0*.*001;* Trial × CS-type*: β* = *0*. *64*, *SE* = *0*.*07*, *t* = *9*.*68*), see Fig. 2. There was no significant CS-type × Group interaction (*p* = *0*.2*6*) or CS-type × Trial × Group interaction (*p* = *0*.2*5*). Our results indicate that even though participants in the Anxious Model group might have had a higher expectancy for the learning model to experience the US, both groups were able to discriminate CS+ from CS− equally well during acquisition, see also Fig. 2.

We further investigated the interaction with STAI-T (STAI-T × Group × CS × Trial). There was no significant interaction with STAI-T and Group (all ps > 0.25) but there was a trend towards a STAI-T × CS-type interaction (*p* = *0*.*056*) driven by slightly worsened CS discrimination in individuals higher in STAI-T.

#### Startle responses

During observational acquisition there were no significant interactions between Group and CS-type, Trial or both (all ps > 0.33). FPS responses were generally higher in the Non-Anxious Model compared to the Anxious Model group (significant main effect of Group *Χ*^2^(*1*) = *5*.*32*, *p* = *0*.*021*; *β* = *0*.*16*, *SE* = *0*.*07*, *t* = *2*.*31*) but there was no main or interaction effect of CS-type (all ps > 0.33). Thus, even though analyses of expectancy ratings showed that participants did learn over time, this learning was not reflected in the FPS. The lack of expressed learning using psychophysiological measures differs from results from previous studies using a similar paradigm^19,27,32,33^. However, all of these previous studies used skin conductance responses to measure fear learning and shocks to the learning model as observational US and it is possible that such methodological differences influenced our results.

### Direct test phase – discriminative learning

#### Expectancy ratings

During the test phase, participants were asked to continuously rate to which degree the believed they themselves were soon about to hear the aversive sound (the US). To investigate discriminative learning, we carried out a set of analyses on expectancy ratings using LMMs that included the CS+ and CS− but not the CSn-. These analyses showed higher expectancy ratings for the CS+ compared to the CS− (significant main effect of CS-type *Χ*^*2*^(*1*) = *45*.*08*, *p* < *0*.*001; β* = *3*.*30*, *SE* = *0*.*52*, *t* = *6*.*34*). There was also a decrease in expectancy ratings over trials (significant main effect of Trial *Χ*^*2*^(*1*) = *67*.*11*, *p* < *0*.*001*; *β* = *−0*.*33*, *SE* = *0*.*069*, *t* = *−4*.*70*), a decrease that was steeper for the CS+ compared to the CS− (significant CS-type × Trial interaction (*Χ*^*2*^(*1*) = *56*.*50*, *p* < *0*.*001; β* = *−0*.*38*, *SE* = *0*.*098*, *t* = *−3*.*91*). Finally, there was also a significant Group × CS-type × Trial interaction (*Χ*^*2*^(*3*) = *4*.*21*, *p* = *0*.*04; β* = *0*.*29*, *SE* = *0*.*14*, *t* = *2*.*05*). Follow up tests with two separates LMMs (CS+/CS−) showed that the Group × CS-type × Trial interaction was driven by a steeper decrease over trials for the CS+ in the Non-Anxious Model compared with the Anxious Model group (Group:Trial| CS+: *β* = *−0*.*14*, *SE* = *0*.*14*, *t* = *−1*.*00*) but a flatter decrease over trials for the CS− (Group:Trial|CS−, *β* = *0*.*15*, *SE* = *0*.*10*, *t* = *1*.*50*) in the Non-Anxious Model group compared with the Anxious Model group, see Fig. 2. This indicated slower extinction of the differential ratings for the Anxious Model group compared to the Non-Anxious Model group. This finding was further confirmed by follow up t-tests of the ratings in the last trial where the Anxious Model group still differentiated between CS+ and CS− (*t*(*67*.*73*) = *2*.*38*, *p* = *0*.*02*, uncorrected alpha) while the Non-Anxious Model group did not (*p* = *0*.*27*, uncorrected alpha).

We further investigated the interaction with STAI-T (STAI-T × Group × CS × Trial). There was no significant interaction between STAI-T and Group (all ps > 0.10) but there was a STAIT × CS-type interaction (*Χ*^*2*^(*1*) = *4*.*17*, *p* = *0*.*04*), resulting from worsened discrimination between CSs in individuals with higher trait anxiety as evident by a positive effect of STAI-T during rating of CS− (*β* = *0*.*069*, *SE* = *0*.*045*, *z* = *1*.*56*) which was attenuated during rating of CS+ (*β* = *−0*.*14*, *SE* = *0*.*056*, *z* = *−2*.*51*) (see also 2.3.1.).

#### Startle responses

Analyses of startle responses during the test phase showed no significant main effect or interaction with Group (all ps > 0.07) and we therefore removed the Group term from further analyses. Further analyses revealed larger FPS for the CS+ compared to the CS− (significant main effect of CS-type *Χ*^*2*^(*1*) = *14*.*84*, *p* < *0*.*001; β* = *0*.*22*, *SE* = *0*.*06*, *z* = *3*.*85*) as well as a decrease in responses over time (significant main effect of Trial *Χ*^*2*^(*1*) = *19*.*46*, *p* < *0*.*001*; *β* = *−0*.*11*, *SE* = *0*.*03*, *t* = *−4*.*41*). Thus, even though the analyses of the FPS responses showed no signs of discriminative learning during the observational acquisition phase it is evident in the direct test phase that participants learned to distinguish between the two CSs. There was no main effect of or interaction with STAI-T on discriminatory learning as measured by startle (all ps > 0.33).

### Direct test phase – generalization

#### Expectancy ratings

To analyse effects of generalization we included the CSn in our LMMs. Analyses of the expectancy ratings during the test phase demonstrated a significant Group × CS-type × Trial interaction (*Χ*^*2*^(*1*) = *6*.*88*, *p* = *0*.*03*). Follow up trend analyses did however not show any difference between groups regarding the quadratic component of the generalization slope (all ps > 0.12), which would indicate to what extent fear or safety learning generalized to the novel CS. Instead, the significant interaction was driven by the difference between groups in the extinction rates of the CS+ and CS−, as reported above (see 2.4.1.). Additional analysis showed no CS-type × Group interaction on expectancy ratings during the first trial only (*p* = *0*.*39*). This further supported our conclusion that there was no difference in generalization between groups.

#### Startle responses

Similarly, there was no significant Group × CS-type × Trial interaction (*p* = *0*.*18*) or Group × CS-type interaction (*p* = *0*.*91*) when we included CSn in our analyses, indicating that there was no difference in generalization between groups. Additional analysis of the FPS during the first trial, before extinction had taken place, showed no CS-type × Group interaction (*p* = *0*.*53*) which further supported this conclusion.

### Post-experiment ratings and questions

After the experiment, participants were asked to answer a set of questions regarding their experience of the experiment and the learning model. Analyses using independent t-tests showed that the Anxious Model group tended to rate the learning model’s experience of hearing the aversive sound as more unpleasant than the Non-Anxious Model group on a seven-level Likert Scale (Anxious Model: Mean = 6.50, SD = 0.60; Non-Anxious Model: Mean = 5.97, SD = 1.39; *W* = *949*, *p* = *0*.*07*). There was further a trend towards the Anxious learning model being rated as more anxious than the Non-Anxious model (Anxious model: Mean = 7.41, SD = 3.13; Non-Anxious model: Mean = 6.27, SD = 2.92; *W* = *949*, *p* = *0*.*07*), according to a subset of questions taken from the GAD 7 scale (range 0–12 points, see Supplementary and Methods for details). A linear mixed effect model with by-participant random intercepts revealed an interaction of Group and CS type (*Χ*^*2*^(*2*) = *20*.*53*, *p* < *0*.*001*) on post-experiment ratings on how worried participants had been to hear the aversive sound during the test phase. Follow up contrasts (Bonferroni corrected) revealed that even though participants in both groups were more worried upon seeing the CS+ compared to both the CS− and CSn (all ps < 0.001) the Non-Anxious Model group reported that they had been more worried upon seeing the CSn compared to the CS− (*β* = *1*.*64*, *SE* = *0*.*38*, *z* = *4*.*30*, *p* < *0*.*001*) while the Anxious Model group did not differ in their level of worry between the two CSs (*p* = *0*.*49*). This could be interpreted as a sign of more efficient safety learning in the Non-Anxious Model group compared to the Anxious Model group, similar to a transferring of anxious behaviour from model to observer.

There were no differences between groups in state anxiety (*p* = *0*.*43*), as measured by STAI-S, and no differences in how unpleasant participants believed they themselves would have experienced the aversive sound (*p* = *0*.*37*).

## Conclusion

We set out to investigate if and how observational fear conditioning is influenced by the expression of anticipatory anxiety in the learning model, and if this could be affected by the observer’s trait anxiety. Interestingly, contrary to our main hypothesis; that participants observing an anxiously behaving model would learn to discriminate less efficiently, analyses of the FPS and expectancy ratings showed that discriminatory learning was more efficient in the Anxious Model group. This was evident by slower extinction of the expectancy ratings for the CS+ in the Anxious Model group compared to the Non-Anxious Model group. In fact, participants in the Anxious Model group, but not the Non-Anxious Model group, still differentiated between the CS+ and CS− at the end of the test phase. Analyses of the post-experimental ratings of the level of worry associated which each CS during the test phase did however indicate worsened safety learning in the Anxious Model group, more in line with our initial hypothesis. As predicted, analyses of the effects of anxiety proneness revealed that higher levels of trait anxiety were associated with lower levels of discrimination between the safe and the threatening stimuli, but this effect did not interact with the anxious behaviour of the learning model.

As for the secondary aim of our study, to develop an observational conditioning paradigm with aversive sound as US, we clearly showed that participants in the current paradigm exhibited successful conditioning. Participants had higher expectancy ratings and larger startle responses to the CS+ compared with the CS−. The paradigm thus appears suitable for use in studies where more traditional USs, such as electric shocks, are not recommended (see also the Discussion for details).

## Discussion

In the present study, we investigated if and how the behaviour of a learning model, either typically anxious or non-anxious, affected observational conditioning, and if this was influenced by the observer’s trait anxiety. Results from analyses of expectancy ratings and startle responses showed that participants learned to discriminate between the safe (CS−) and unsafe (CS+) stimuli equally well although there was a slower extinction of the expectancy ratings for the unsafe stimulus in the group that observed an anxiously behaving learning model. We thus found no evidence to support our hypothesis that discriminatory learning should be worsened following observation of an anxious, rather than a non-anxious, learning model. On the contrary, we found signs of more robust discriminatory learning during observation of the anxious model, expressed as slower extinction. In fact, expectancy ratings during the last trial in test phase showed that participants in the Anxious Model group still differentiated between the CS+ and CS− although the participants in the Non-Anxious Model group did not. One explanation of these results could be that the learning model’s anxious behaviour was interpreted as a response to a highly aversive US (or dangerous context), which could make learning more relevant^15^, and thus more resistant to extinction. This explanation was to some extent supported by the group difference in expectancy ratings during observational acquisition, where the Anxious Model group had a higher expectancy overall that the learning model would hear the aversive sound (the US), i.e. a more dangerous context, as compared to the Non-Anxious Model group. In addition, participants in the Anxious Model group estimated that the learning model experienced the US as more aversive than participants in the Non-Anxious Model group. Another possibility is that the aversive sound (the US) is perceived as more unpredictable in the Anxious Model group, due to the non-discriminatory responses to the CSs, and that this experience of unpredictability leads to slower extinction. Such an explanation would be related to the resistance to extinction effect resulting from partial reinforcement schedules^34^. We did not see any interaction between the participants’ trait anxiety and the expressed anxiousness of the model. It is possible that the variance of trait anxiety was too narrow in our sample to capture any effects. Although our findings are interesting, we are cautious in our interpretation of the results. The study shows that typically anxious anticipatory behaviour in the learning model does influence observational learning even though it is clear that more research is needed to be able to understand how others’ expression of learned fear influences observational conditioning.

The secondary aim of the study was to develop an observational fear conditioning paradigm using auditory US/threat of auditory US. Our results clearly showed that the paradigm presented here is suitable for this purpose. However, similar to the observational conditioning paradigm^29^ that the current design is based on, participants here never experienced the US directly. This became slightly problematic since we also used a sound, a burst of noise, as a startle probe. We therefore had to make it very clear for the participants that the startle probe and the aversive sound that the learning model heard were two different sounds. Using skin conductance responses (SCR) to measure learning would eliminate this problem (although note that SCR and FPS are not equivalent^21^). The fact that the participant never experiences the US generates a second problem related to our paradigm, namely that it likely increases the extinction rate during the test phase. In the influential generalization paradigm developed by Lissek and colleagues^35^, the US is still presented during the generalization test but with reduced probability (50% reinforcement schedule in the test phase compared to 75% in the acquisition phase). This leads to a reduced extinction rate allowing for a very long test phase with a total of 72 CS presentations, twice the number of presentations in the present study. For future studies, unless US presentations are introduced in the test phase, reducing the number of CS presentations might be desirable.

In the present study, it was clear to the participants when the learning model experienced the aversive sound (the US). This was done to be able to study the effects of anticipatory responses in addition to and not instead of, the direct responses to the US. However, it also limits the generalizability of our study. Thus, our results do not generalize to situations where only information of anticipatory responses, not direct consequences, is given. We consider the present study to be a first step in investigating the effects of a learning model’s anticipatory responses on observational fear learning. For the future, it would be interesting to combine a learning model’s anticipatory responses with verbal information of which stimulus is a threat and which is safe. Investigating the effect of anticipatory responses on observational learning is important, mainly because of the frequency with which we expect them to be present in real life compared to the frequency of observing other’s direct responses to aversive stimuli. From a clinical perspective, it is also important to investigate if anxious behaviour can be transferred from a learning model to an observer, for instance from an anxious parent to an anxiety prone child^36^.

## Methods

### Participants

Eighty-three participants were recruited and paid for participation in the experiment approved by the Regional Ethical Review Board in Stockholm and the experiment was performed in accordance with relevant guidelines and regulations. All participants signed an informed consent form before the experiment. Two participants were excluded from further analyses due to misinterpretation of the instructions, one was excluded based on participation in a similar study and one was excluded due to a technical error during data collection. The remaining seventy-nine participants were randomized into two groups: the Non-Anxious Model group (n = 39, 16 male; Mean age = 26.7 y, SD = 5.5 y) who observed a demonstrator expressing fearful anticipatory responses upon seeing an unsafe stimulus, the CS+, but not when watching a safe stimulus, the CS− (see Material for details), and the Anxious Model group (n = 40, 14 male; Mean age = 26.4 years, SD = 4.9 y) who observed a demonstrator expressing fearful anticipatory responses upon seeing both CSs (see 5.2. Material for details).

### Material

Three coloured squares served as conditioned stimuli, CSs. To avoid generalization of learning based on colour similarity the colours had the same saturation and luminance and were equally distanced to each other in hue (orange: 20°, green: 140°, purple: 260°). The stimuli used during the observational acquisition phase consisted of videos edited using Adobe Premiere Pro CS5.5. The videos depicted a learning model seated in front of a computer screen upon which the CS+ and CS− were presented, while wearing headphones. The CS+ occasionally co-terminated with an 6 s long aversive sound (number 244, the sound of a man making a wheezing sound almost as if choking) from the IADS (International Affective Digital Sounds) collection^31^ presented in the headphones of the learning model and not perceivable to the participant. The CS− was never paired with the aversive sound. The learning model reacted negatively when hearing the sound and was instructed during recording of the videos to respond as if not wanting to hear the sound and to respond in the same way during each presentation of the sound. To clarify when the aversive sound was presented a speaker symbol was shown on the screen when the sound was presented to the learning model. In the video used in the Non-Anxious Model group, the learning model reacted with a slightly negative anticipatory expression upon seeing the CS+ on all presentations following the first CS+ presentation paired with the aversive sound. In the video used in the Anxious Model group, the learning model reacted with the same slightly negative anticipatory expression but this time upon seeing all CS presentations, both CS+ and CS−, following the first CS+ presentation paired with the aversive sound. The same learning model was used for both videos. For the anticipatory response he was instructed to react to the CS as if getting ready for or anticipating an aversive experience, essentially by squinting his eyes and frowning a bit. For the direct aversive response he was instructed to react as if not wanting to hear the sound, by raising the eyebrows, frowning more and moving the head slightly backwards. The learning model was instructed to always react in the same way and to make the shifts between expressions natural but clear and to try to go back to the same neutral expression in between the aversive responses. CS presentations were counterbalanced to cover all possible colour combinations. We further combined two different CS presentation orders during the observational acquisition (most importantly differing in which CS was presented first) with three different CS presentation orders during the test phase (most importantly differing in which CS was presented first). This resulted in six different CS presentation orders. As startle probes we used 50-ms bursts of 95-dB white noise with a near instantaneous rise time (<1 ms).

### Data acquisition and preprocessing

Throughout the whole experiment, participants were asked to use a slider to rate their expectation that the aversive sound was about to be played, either so that they themselves would hear it (during pre-acquisition and the direct test phase) or the observed learning model (during observational acquisition). Ratings ranged from “not at all likely” to “very likely”. We collected ratings of the expectation that the aversive sound would be presented to either the learning model or themselves on the CS presentations that were not startled.

Startle blink was measured by recording electromyographic (EMG) responses of the left orbicularis oculi muscle using two Ag/AgCl electrodes prepared with electrolyte gel, sampling rate 1000 Hz. A ground electrode was placed on the forehead. Startle probes were presented binaurally through headphones on half of the CS presentations such that for every pair of two consecutive presentations of the same CS, one of these CSs and one related ITI was startled.

Data from the expectancy ratings and EMG electrodes were recorded using BIOPAC Systems (Santa Barbara, CA) and preprocessed using AcqKnowledge software (BIOPAC Systems Inc., California). The expectancy ratings for each CS and trial was calculated as the mean rating over the whole CS presentation using the continuous ratings. EMG data were amplified and filtered with a 28–500 Hz IIR band pass followed by a 50 Hz IIR band stop. Data was then integrated using a time constant of 20 ms. The startle response was calculated as the difference between a baseline measured as the mean response in the 50 ms preceding the startle probe and the maximum response within a 100 ms time window starting 20 ms after the onset of the startle probe. Baseline comparison of startle responses was carried out on ITI measures using linear mixed models with Group and Trial as predictors (see also Supplementary). This clarified that there were no main or interaction effects of Group on startle responses during ITI for either pre-acquisition, observational acquisition or test phase (all ps > 0.27). Startle responses for the CSs at each trial were then baseline corrected using the corresponding startle response at ITI.

### Procedure

The experiment consisted of three phases, the pre-acquisition phase, observational acquisition phase and test phase. During the pre-acquisition and observational acquisition phase two CSs, CS+ and CS−, were presented; directly on the screen in the pre-acquisition phase and on the screen observed by the learning model in the video in the observational acquisition phase. (For an overview, see Fig. 1.) In the test phase, a novel CS, CSn was introduced to measure generalization of learning. The pre-acquisition phase consisted of 4 presentations of each CS (2 startle trials, 2 expectancy ratings trials), the observational acquisition phase and test phase consisted of 12 presentations of each CS (6 startle trials, 6 expectancy ratings trials). CS presentations were pseudorandomized so that one startled CS of each type and one rated CS of each type were grouped together in blocks. Within each block, one ITI was startled and this measure was later used as baseline. Of the 12 CS+ presentations in the observational acquisition phase 9 (75%) were followed by a 6 s long unpleasant sound played in the headphones of the learning model (the learning model’s US, not accessible to the participant) accompanied by the learning model’s expressed aversive response and the presentation of a small loudspeaker symbol on the screen to clarify that the learning model now heard the aversive sound. In addition to reacting to the aversive sound, the learning model in the Anxious Model group reacted aversively to the presentation of both the CS+ and CS− while the learning model in the Non-Anxious Model group reacted aversively to the presentation of the CS+ only (see also *5*.*2*. Material). The procedure for the participants in the Non-Anxious Model and the Anxious Model group differed only in the video used for the acquisition phase.

Participants were instructed that they during the experiment were to observe presentations of coloured squares and that immediately following some presentations they would hear an approximately 6 s long aversive sound, different from the short burst of noise used as a startle probe. Before the pre-acquisition phase participants were told that they would not hear the aversive sound during this phase but only observe the coloured squares. They were instructed that the video in the acquisition phase they were to observe depicted a person going through a similar experiment as the one they were about to experience, observing the same coloured squares and hear the same aversive sound. Following the experiment, participants were debriefed and filled out two questionnaires: the State-Trait Anxiety Inventory^37^ and the Balanced Emotional Empathy Scale^38^. To asses the degree to which the participants evaluated the learning model as anxious we asked them to rate the learning model according to four questions based on the GAD-7^39^, a tool used to briefly assess general anxiety disorder but here modified for the purpose of assessing perceived anxiousness (for details see Supplementary).

### Analyses

Analyses were carried out using R^40^. All linear mixed models (LMMs) included by-participant random intercepts and random slopes for all within-subject effects^41^ (unless convergence issues forced us to remove them). We modelled Group, CS-type and Trial as fixed effects. CS-type included two levels (CS+, CS−) when we tested discriminatory learning and three levels (CS+, CS−, CSn) when we tested generalization effects. Reported main and interaction effects are based on the type II Wald test. For all analyses an alpha level of 0.05 was used.

## Supplementary information

Supplementary Information

## Data Availability

The datasets generated and analysed during the current study are available from the corresponding author on reasonable request.
