# Supplementary material for: Anxious behaviour in a demonstrator affects observational learning
Source: Sci Rep. 2019 Jun 24;9:9181. doi: 10.1038/s41598-019-45613-1 (PMC6591253; doi:10.1038/s41598-019-45613-1)
Supplement: Supplementary file 1 — Supplementary Information [file 41598_2019_45613_MOESM1_ESM.pdf]

Supplementary Information:

Anxious behaviour in a demonstrator affects observational learning

Ida Selbing[1*], Andreas Olsson[1]

[1] Division of Psychology, Karolinska Institutet, 171 77 Stockholm, Sweden

[*] To whom correspondences should be addressed: idaselbing@gmail.com

Assessing a rating of the learning model's perceived level of anxiety

After the experiment, participants were asked to rate how they perceived the learning model based on four questions taken from the GAD-7 (Spitzer, Kroenke, Williams, & Löwe, 2006), a scale developed to measure generalized anxiety disorder, which we modified for our purposes, i.e. framed so that it concerned someone else, rather than the responder. Participant were asked to rate on a scale from 0 (doesn't fit at all) to 3 (fits well) how well they though the following descriptions fit the person in the movie (i.e. the learning model). They were told that there were no right or wrong answers and that they should try to answer according to their impressions:

1. He often feels nervous, anxious or on edge.
2. He often worries too much about different things.
3. He often has trouble relaxing.
4. He often feels afraid as if something awful might happen.

The total score was calculated as the sum of the answers to all four questions.

Startle baseline

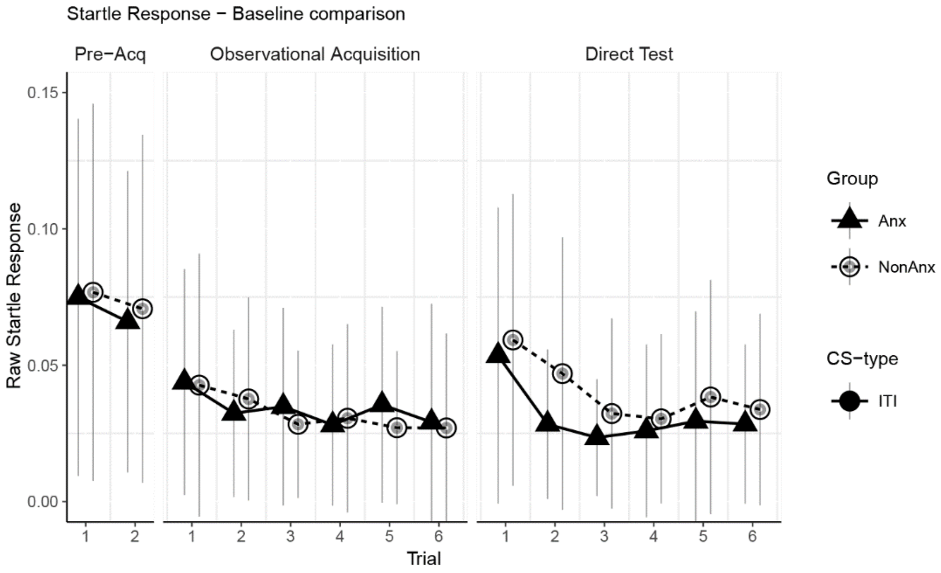

The raw startle response measured at ITI used as baseline plotted for the three phases. Baseline comparisons revealed no group differences.
